# Importance of Ecological Variables in Explaining Population Dynamics of Three Important Pine Pest Insects

**DOI:** 10.3389/fpls.2018.01667

**Published:** 2018-11-13

**Authors:** Rainer Hentschel, Katrin Möller, Aline Wenning, Annett Degenhardt, Jens Schröder

**Affiliations:** ^1^Faculty of Forest and Environment, University for Sustainable Development, Eberswalde, Germany; ^2^Brandenburg State Forestry Center of Excellence, Eberswalde, Germany; ^3^Thünen-Institute of Forest Ecology, Eberswalde, Germany

**Keywords:** ecological modeling, Random Forest, mass outbreak, *Pinus sylvestris*, *Lymantria monacha*, *Dendrolimus pini*, *Diprion pini*

## Abstract

Climate change challenges forest vitality both directly by increasing drought and heat periods and indirectly, e.g., by creating favorable conditions for mass outbreaks of phyllophagous insects. The large forests dominated by Scots pine (*Pinus sylvestris* L.) that cover the lowland regions in northeast Germany have already been affected regularly by cyclic mass propagations of defoliating insect species in the past with climate projections implying an even more advantageous environment for devastating outbreaks in the future. To improve predictive and responsive capacities we have investigated a wide range of ecological parameters to identify those most strongly related to past outbreak waves of three central species. In total, we analyzed 3,748 variables covering stand and neighborhood properties, site quality, and climatic conditions for an area of roughly 750,000 ha of pine forests in the period 2002–2016. To reflect sensitivity against varying climate, we computed “floating windows” in relation to critical phenological phases of the respective insects. The parameters with the highest explanatory power resulted from the variable importance measures of the Random Forest (RF) methodology and have been evaluated by a 10-fold cross-validation process. Our findings closely reflect the known specific gradation patterns and show that relative variable importance varies with species. While *Lymantria monacha* L. feeding was mainly dependent on the surroundings of the respective stand, *Diprion pini* L. proved to be almost exclusively susceptible to climatic effects in its population dynamics. *Dendrolimus pini* L. exhibited a mixed pattern of variable importance involving both climatic and forest structure parameters. In many cases the obtained statistical results support well-known ecological cause-effect relations and long-term population change dynamics. The RF delivered very high levels of sensitivity and specificity in the developed classifications and proved to be an excellent tool to handle the large amounts of data utilized for this study. While the presented classification approach may already support a better prognosis of the amplitude during the outbreak culmination, the obtained (most important) variables are proposed as preferable covariates for modeling population dynamics of the investigated insect species.

## Introduction

Climate change is challenging forest management strategies around the globe (Kirilenko and Sedjo, 2007; Lindner et al., 2014). In addition to the expected detrimental effects of increasing heat and drought on major physiological processes of forest trees (Allen et al., 2015), the potential stimulation of mass outbreaks of thermophilic forest pest insects is threatening sustainable forest management (De Lucia et al., 2008; Jactel et al., 2012).

**FIGURE 1:**
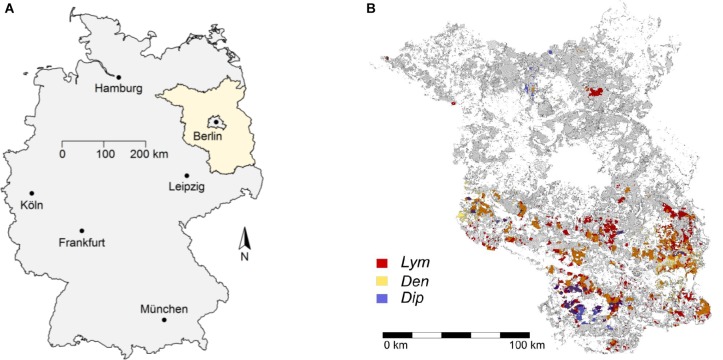
The federal state of Brandenburg is located in the Northeast of Germany **(A)**. The 40,005 forest compartments (FC) in the state are shown in gray shading **(B)**. FC affected by PPI defoliation are filled with the color code of nun moth (*Lym* = red), pine-tree lappet (*Den* = yellow), and pine saw fly (*Dip* = blue). Outbreaks of more than one species in identical FC are indicated by divergent shading (e.g., orange colors show FC affected by *Lym* and *Den*).

The federal state of Brandenburg, Germany, has been prone to severe damages in Scots pine (*Pinus sylvestris* L.) stands by defoliating forest pest insects for a long time (Figure 1). Around 75% of the forest area in Brandenburg (ca. 1,000,000 ha) is dominated by Scots pine which, particularly in even-aged pure stands, provides favorable feeding conditions for phytophagous pine pest insects (PPIs). Due to the frequent occurrence of mass outbreaks (Gräber et al., 2012), the forest protection service responsible for the Brandenburg area has been operating a comprehensive monitoring program for individual pest insects tracking their population dynamics since the beginning of the 1930s. Experience has shown that mortality sharply increases if needle losses due to feeding by PPI exceed a threshold of 90% (Wenk and Möller, 2013). In cases of defoliation prognosis of this dimension, selective insecticides are applied to prevent the total loss of the affected forests and their functions. Massive feeding damage causes major disruptions of ecosystem relations, which in turn may lead to long-term changes of forest structure (Otto, 1994).

Three of the most important PPI more or less regularly infesting large forest areas in northeast Germany are nun moth (*Lymantria monacha* L., “*Lym*”), pine-tree lappet moth (*Dendrolimus pini* L., “*Den*”), and pine saw fly (*Diprion pini* L. “*Dip*”). Specific monitoring and counteractive algorithms have been set up in reaction to their different biology and outbreak behavior (Böhme and Haffelder, 1999; Hielscher and Engelmann, 2012; Möller et al., 2017). While *Den* and *Dip* are feeding exclusively on *Pinus sylvestris, Lym* is a polyphagous species. Its larvae may develop, in addition to most conifer species, on many deciduous trees and shrubs. In the northeast German lowlands, however, destructive mass attacks tend to be limited to pine stands (Häußler et al., 2000; Hielscher and Engelmann, 2012).

Comparing the historical outbreaks of pine pests in the northeast German lowlands, Gräber et al. (2012) describe a relatively uniform frequency (8–12 years) of *Lym* gradations, while mass outbreaks of *Den* have increased in frequency with climate change. *Dip* shows a great irregularity in the timing of gradations over the observed period 1920–2013. Ray et al. (2016) and Schafellner and Möller (2018) described the preference of *Den* for warm and dry late summer months and classified this species as a climate-change winner. For *Dip* the observation of a new unexpected and strong mass outbreak in northeast Germany in 2016 was the reason to investigate the causal relations between weather and population development in detail (Möller et al., 2017).

In Brandenburg, 11 mass propagations have been documented for *Lym* between 1922 and 2010 (Gräber et al., 2012). The life cycle of the species in the region starts with the larvae developing in their eggs already in early fall. They hibernate in this form at the base of the tree trunks and hatch after warm periods in April or May (Schwenke, 1978). Significant needle feeding starts with the L2/3 stage (= second and third larval development stage) and lasts until pupation in June. A few weeks later the imagines appear and begin to mate; the females deposit their eggs immediately afterward, usually in coarse bark fissures in the lower part of the tree trunks. Warm and dry conditions, for example during the mating period, promote individual and population development (Zwölfer, 1935; Häußler et al., 2000).

The pine lappet moth has also been a potential threat to Scots pine forests in the region for a long time (Schwenke, 1978). The species is more dependent on climatic triggers in the timing of mass outbreaks than *Lym*, their frequency has increased throughout the past decades (Gräber et al., 2012; Schafellner and Möller, 2018). The first instar larvae hatch in late summer from eggs usually deposited at needles and twigs in the pine crowns and start to feed until the first frost events force them to climb down and enter hibernation in the upper layers of the forest soil. In early spring the L3/4 instars return to the crowns and resume feeding. Pupation occurs from June to July; the adults emerge shortly afterward and mate with each female laying up to 300 eggs (Schwenke, 1978; Möller et al., 2007). High population densities during mass outbreaks lead to extreme defoliation covering the complete age range of the host trees with heavy feeding even of juvenile plants. Additionally, caterpillars tend to consume all green parts of the host including buds and needle sheaths (Weckwerth, 1952; Möller and Engelmann, 2008; Schafellner and Möller, 2018).

The life cycle of *Dip* is very complex and may change from univoltine to bivoltine patterns in Brandenburg. Large-scale defoliation events are usually coupled with bivoltine years with the second generation feeding in fall. Massive damage and widespread tree mortality may occur in these years because the first generation larvae feed on needles from the previous year and the second generation on current year needles. A detailed description of the biology and of the frequency and consequences of mass outbreaks is provided by Möller et al. (2017). Among the three selected PPI, *Dip* seems to show the largest dependency on climatic factors with warm and dry summer periods contributing significantly to mass outbreak probability (Geri, 1988; Möller et al., 2017).

Scientific analyses and practical experience have shown that population dynamics of the PPI are partly characterized by cyclic patterns leading to mass gradations of a more or less stable frequency (Schwenke, 1978; Altenkirch et al., 2002). On the other hand, the exact timing, the extent, and the consecutive damage of outbreaks are shaped by abiotic factors, mainly weather conditions (Möller et al., 2017). The latter influence population dynamics directly (as favorable or disadvantageous environments) and indirectly by affecting the host plant (Sierpiñska, 1998; Breda et al., 2006). These indirect effects – together with the gradation history of the respective forest stands – shape the predisposition of trees and forests toward mass outbreaks. The complexity of these relationships is further aggravated by climate change processes which affect both the physiological composition and predisposition of the forests and the population dynamics of the PPI (Jactel et al., 2012).

Against this background, there is an increasing need to explore the causal relationships of forest pest insects’ population dynamics to environmental drivers in order to enhance the existing monitoring programs, to reduce insecticide treatments to a minimum and to develop forest management strategies accounting for biotic risk prognosis. Furthermore, the climate sensitivity of individual pest species needs to be contrasted to the forest development at landscape level which in turn affects the forests’ predisposition and their quality as source of feeding and breeding ground for these insect species and their antagonists.

This study aims to determine the most important influencing factors controlling mass outbreaks of nun moth, pine-tree lappet moth, and pine saw fly in Scots pine forest ecosystems. For this purpose, we applied the “Random Forest” approach (RF; Breiman, 2001) to an extensive database covering the forests in the federal state of Brandenburg. Focusing on the variable importance measures of the RF methodology, we performed a variable selection procedure highlighting the most explanatory variables representing climatic conditions (clim_), site properties (site_), forest stand structure (stand_), and forest landscape description (forest_). The variable selection process, hence, provides a set of covariates most suited for modeling future feeding hot spots under consideration of stand development and climate change.

We hypothesize that (a) the PPI are sensitive to independent climatic triggers and temporal periods within the year relating to their particular biology. Furthermore, we propose that (b) the preferences to particular stand structures vary for the three PPI due to their specific adaptation to ecological (sub-) niches of pine forest ecosystems. Based on the relevant literature and practical experiences we assume that (c) PPI share a common preference for warm climate and that (d) a higher tree species diversity of the habitat is counteracting severe mass outbreaks due to the limiting effects exerted by the higher abundance of predators and parasitoids in such conditions.

## Materials and Methods

The database of this study was built as a comprehensive set of ecological variables available for almost the total forest area in Brandenburg for the years 2002–2016. This area is subdivided into “forest compartments” (FCs), which is the basic administrative unit of forest services in Germany. In Brandenburg, the forest area is organized into a total number of 40,005 FC (reference year 2014). The ecological variables of these plots were computed for different statistical metrics, weighting factors and temporal aggregates. We generated a total of 3,748 variables in a first step and condensed our analysis to a few variables of largely independent information at the end of the study. All analysis steps have been performed in “R” (R Core Team, 2014) and we used the ‘randomForest’ package (Liaw and Wiener, 2002) stressing the most important variables of PPI mass outbreaks.

### Defoliation Assessment

Based on forest damage assessments of the years 2002–2016, the forest protection service of Brandenburg provided feeding statistics of the PPI for all FC affected by defoliation during this period. The spatial distribution of the historical defoliation areas is shown in Figure 1B. The forest area of Brandenburg is displayed by gray shading. Around 75% of all FC feature Scots pine as the leading tree species. This amounted to an area of approximately 750,000 ha of potential feeding ground for the PPI. Defoliation damages were concentrated in the South of Brandenburg except for feeding events of *Lym* in 2003 and of *Dip* in 2005.

**FIGURE 2:**
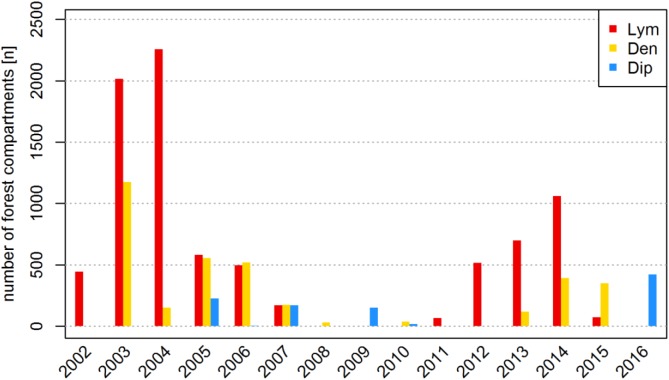
Number of forest compartments (FC) affected by defoliation by *Lym, Den*, and *Dip* in Brandenburg from 2002 to 2016.

The terrestrial forest damage assessment in Brandenburg includes the area and the relative intensity of defoliation and the involved species of PPI. Feeding activities are documented by the local foresters who are in charge of 15,000 ha forest area on average, so defoliation events of low feeding intensity might have been missed. Another source of inaccuracy is introduced by airborne pesticide applications (PAs). Since PAs are restricted to FC predicted by the state forest protection service to become completely defoliated based on the related monitoring data and the specific regional population dynamics, we considered those FC as representing total defoliation. In Figure 2, the population dynamics of the three studied PPI is illustrated by the number of FC affected by defoliation over time.

### Influencing Factors

We gathered an extensive and diverse set of potential influencing factors partitioned into climatic conditions (clim_), site properties (site_), stand description (stand_), and the forest structure within a circular buffer zone of 1,000 ha (forest_). In respect to these variable groups we computed various temporal compositions of climatic parameters and different aggregates of the forest characteristics based on their spatial abundance. We thus obtained a total number of 3,748 variables. A tabular description including an encoding scheme for the different variable groups is presented in the Supplementary Tables 1–4. The variable coding always starts with the acronym of the variable group and the accessed parameter, followed by further specification of computation routine. Important variables, however, have been expressed verbally within this article.

#### Climatic Conditions (clim_)

The German Meteorological Service (DWD) provides daily measurements of various climate parameters across a comprehensive network of weather stations in Germany. Köhler et al. (2015) made use of these data in a regionalization approach providing 100 m × 100 m grids of relevant climate data used in this study (Supplementary Table 1). Information on potential evapotranspiration was modeled according to Penman–Monteith (see Allen et al., 1998). Except for the precipitation data, all parameters were simulated using a generalized additive model (GAM; Wood and Augustin, 2002). Precipitation data were regionalized using an ordinary Kriging approach (Bivand et al., 2013).

According to the respective grid built by the center points of all FC, daily climate data were summarized to temporal aggregates of all available climatic parameters (Supplementary Table 1, second position). Furthermore, our investigation includes intervals attached to varying phenological dates rather than being fixed to a specific day of the year (DOY). In consequence, we estimated the beginning and end of crucial life stages of the respective populations. In addition, we tested conventional climatic windows such as annual and monthly aggregates and averages.

Based on the close temporal synchrony between host development and insect phenology we used the modeled day of bud burst (BBT) for *P. sylvestris* according to the phenological models developed by Menzel (2003) as an indicator of the specific climatic conditions in a given year. Annual dates of BBT usually vary between DOY 118 and 128 which translates to April 28 and May 8, respectively. In addition, we included periods related to the annual dates of BBT plus 90 days (BBT+90 = FLY), which translates to a range between July 27 and August 6, as physiologically important time windows for pupation. This period is particularly relevant for *Dip* because the species may switch into a bivoltine lifecycle with wasp hatching and swarming occurring in the weeks around FLY (Möller et al., 2017).

**FIGURE 3:**
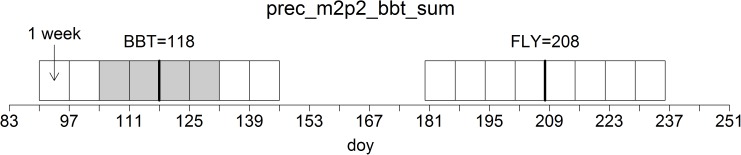
Construction of temporal variables of the selected climatic parameters. In this exemplary case, the variable with the acronym “tmax_fly_m3p3_sum” represents the sum of daily maximum temperatures in a period beginning 3 weeks prior to and terminating 3 weeks after the day of year (DOY) 90 days after modeled bud burst (BBT) of *P. sylvestris*. The potential temporal range of variables is shown as weekly rectangles; the chosen time window is shaded in gray.

In relation to the phenological dates of BBT and FLY, we created factors for periods of at least 1 week covering all possible combinations of weeks before and after the respective dates, starting 28 days before and ending 28 days after BBT and FLY (see Figure 3 for a visual example).

#### Site Properties (site_)

Similarly to the climate data, the “DSS-RiskMan” project provided regionalized raster data of basic site properties in 100 m × 100 m grids. Covariates for the regionalization process originated from geological and edaphic maps, a digital elevation model, long-term climate data, and information about the present forest types (Riek and Russ, 2014; Russ, 2015). Digital soil mapping (e.g., McBratney et al., 2003) was applied providing soil types and soil properties for the usage of appropriate pedotransfer functions (Renger et al., 2009).

Basic soil characteristics were introduced into the analyses as nutrient supply (nv_), actual field capacity (afc_), and plant available soil water (aws_). The latter parameter represents for the present forest type and the specific rooting depth. Furthermore, the long-term evapotranspiration rate (pet_) of the site has been computed according to Penman–Monteith model (see Allen et al., 1998) and a 30-year average of the respective climatic input variables. Considering the present forest type and the degree of stocking resulted in the long-term actual evapotranspiration rate (aet_) (Supplementary Table 2).

#### Stand Description (stand_)

Based on the state forestry inventory, each forest stand in Brandenburg should be gauged in a regular 5–10 years rotation by the angle-count sampling method. Basic stand information about, e.g., basal area and timber stock separated by tree species and stand layer are gathered in the Brandenburg forest database “DSW^2^” which is updated annually with data covering forest growth, forest management activities, and calamity events.

Unfortunately, the support of the “DSW^2^” database has been suspended by non-governmental forest owners since 2007. Hence, as of 2008, for around 73% of the forest stands in Brandenburg (i.e., the share of non-state owned forests) data are available only for mean age, diameter, height, and site index of the present tree species and layers. For these stands, we used yield-table references for pure forest stands in Brandenburg to estimate basal area (m^2^ ha^-1^) and standing timber volume (m^3^ ha^-1^). If information about stand density was missing, estimates of the basic stand parameters had to be assigned to fully stocked stands (stand density index = 1.0) introducing a not-quantifiable bias about the actual basal areas and forest stocks. This data completion was nonetheless necessary to target mass outbreak events of PPI on landscape level in consideration of the spatial correlation between feeding events and forest properties.

**FIGURE 4:**
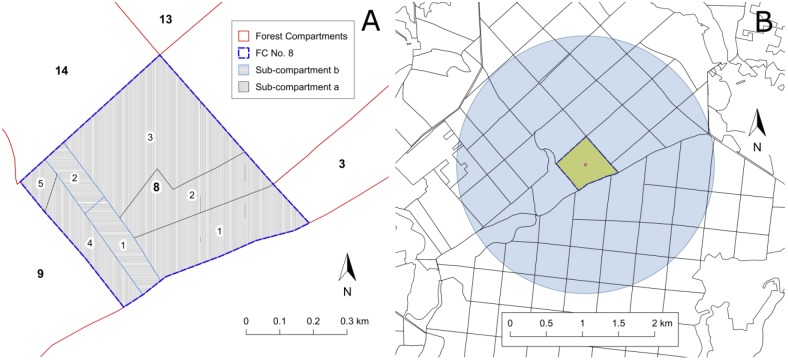
Example of a FC (No. 8) subdivided into sub-compartments (a, b), sub-areas (1–5, 1–2) and management units which may contain several forest stands **(A)**. Individual stand properties are averaged to describe the “stand_” properties of each FC. **(B)** Forest compartment (yellow) with buffer circle of 1,000 ha (blue) analyzed to derive the forest_ properties.

An additional step of data homogenization was necessary because this study focused on the level of the FC and not on the individual forest stands. Since the FC may contain up to 20 smaller management units and forest stands (Figure 4A), we had to aggregate individual stand data into a unique “average” description of the FC (stand_). All considered stand parameters have been weighted by the relative contribution of all forest stands within the FC. Stand parameters and weighting factors are probably biased due to differences in coverage and quality of the forest inventory data. We nevertheless suggest that the general distribution and abundance of forest characteristics within the FC can be preserved and that important influencing factors of PPI rather depend on the forest area properties on a larger scale rather than on detailed single stand properties.

In order to reduce the loss of information within the aggregation process, all stand_ variables were calculated for different tree types (angiosperms and gymnosperms) and tree species (pine and oak) additionally. We also computed the relative proportion of the forest area stocked by different mixture types, tree layers, and age classes. Two additional diversity indices (Shannon and Weaver, 1949; Simpson, 1949) were included which focus on the relative abundance of the respective tree species.

The aggregation of the forest inventory data resulted in 910 variables comprising the arithmetic means and standard deviations of classical stand parameters as well as forest area proportions and forest mixture types at the FC level. The encoding of the considered stand_ variables can be found in Supplementary Tables 3, 4.

#### Forest Structure (forest_)

In order to provide additional information about the neighboring forest stands and the surrounding of the individual FC, the computation routines developed for stand_ variables were adapted for circular buffer zone covering approximately 1,000 ha (Figure 4B). Each FC intersecting a circle with 1,784 m radius around the center of the focus FC was included in the calculation of respective forest_ variables. Except for the separation for pine and oak stands, Supplementary Tables 3, 4 have been applied accordingly. The pre-processing resulted in 560 forest_ variables summarizing the forest inventory data of several hundred stands for one buffer zone.

### Variable Importance

In order to detect the most explanatory variables on the presence of a defoliation event, we applied the RF approach for classification problems introduced by Breiman (2001). Its integrated variable importance measure provides a ranking of the variables according to their explanatory power (see Calle and Urrea, 2011). In fact, the RF has been stated to be the best classifier in comparison to a total number of 179 classifiers tested for “Average Accuracy” and “Friedman Ranking” (Fernández-Delgado et al., 2014).

We used the ‘tuneRF’ function implemented in the ‘randomForest’ package (Liaw and Wiener, 2002) for determination of the optimal number of predictors used for the random variable selection in the construction of the individual decision trees. Our default setting comprised of 1,000 decision trees. All RF were trained by a balanced training data set (see Chen et al., 2004). The validation on the classification performance, however, has been carried out by a 10-fold cross-validation (see Rodríguez et al., 2010) for classification of the entire data set (40,005 observations per year).

In a first step, we studied the variable importance of the individual variable groups and to eliminate non-important and highly correlated variables from the groups. Therefore we constructed three RF according to the three variable groups clim_, stand_, and forest_ (RF_clim_, RF_stand_, RF_forest_). The site_ variables were considered in a later step. After computation of the average “mean decrease Gini” (MDG) based on the 10-fold cross-validation process (RF trained with 90% of the database and tested against the remaining 10%), we excluded variables of small importance as detected by falling below the standard deviation of the average MDG. We also excluded highly correlated variables (Spearman’s rho > 0.9) in an iterative way keeping only the top-ranked variable. This was done although (as shown by Genuer et al., 2010) the relative importance between two variable groups could be preserved and important variables could be distinguished reliably from noise even for a high number of correlated variables. In our case, the applied pre-selection of important and “poorly” correlated variables should preserve the most important variables regardless of redundant data as introduced by variable generation.

In a second step, we analyzed the variable importance for mixed variable groups by means of an additional RF (RF_all_) based on the reduced variable sets and the site_ variables. All previous steps of cross-validation, ranking, and variable elimination were repeated accordingly. This step was performed in order to further distinguish between the variable importance of different variable groups.

As an alternative approach we further eliminated highly correlated variables and condensed the available information to a small set of influencing factors of PPI. This was done in order to obtain a reduced variable set for both interpretation and prediction issues. Therefore, we applied the variable elimination process described by Genuer et al. (2015) on the same data as for RF_all_. In contrast to the former approach, we here included the geographic coordinates (x = easting, y = northing; UTM ETRS89) of the FC in order to account for the spatial distribution of the feeding plots. Ordered by rank according to “mean decrease accuracy” (MDA), the algorithm inserted the variables step-wise into a nested model for classification. Variables are thus kept or eliminated from the nested model according to a threshold of the minimum error gain relating to the “out-of-bag” error (OOB). The threshold was calculated by the mean of the first-order differentiated OOB errors (see Genuer et al., 2015). Using the ‘VSURF’ package (Genuer et al., 2015), we provided an additional variable selection suggested for prediction (RF_V SURF_).

## Results

The figures documenting the importance of the analyzed parameters use different colors for each of the studied pest insects (*Lym* = red, *Den* = yellow, *Dip* = blue). The encoding of the variable names can be found in the supplementary material and is also verbally expressed throughout the following passages. We compared the performance of the RF approaches on different variable selections and obtained the most important variables classifying non-defoliated (*y* = 0) and defoliated (*y* = 1) FC.

The five RF approaches strongly differed in the number of variables used (n_var_) but showed a generally high hit ratio of the defoliation observations (TNR) with at least 70% and an even higher hit ratio of the non-defoliation observations (TPR) with at least 86% (Table 1). RF_V SURF_ applied only a small number of variables and obtained a very high TNR of at least 94%. This is partly due to the slightly different methodology behind RF_V SURF_ which uses MDA as importance measure minimizing the loss of accuracy in the variable elimination process.

**Table 1:** Input data and statistical output according to the five different RF approaches investigating the variable importance toward the feeding events of three PPI.

	PPI	n_y_ =_0_	n_y_ = _1_	n_var_	TPR	TNR	OOB
RF_clim_	*Lym*	70269	8443	2264	98%	80%	3.80%
	*Den*	49501	4428	2264	99%	89%	1.60%
	*Dip*	8527	797	2264	96%	98%	3.60%
RF_stand_	*Lym*	515854	7642	910	86%	79%	14.00%
	*Den*	517636	2800	910	87%	82%	13.10%
	*Dip*	519664	772	910	89%	70%	11.50%
RF_forest_	*Lym*	515854	7642	560	92%	93%	8.20%
	*Den*	520647	2849	560	93%	94%	7.20%
	*Dip*	522690	806	560	96%	92%	3.60%
RF_all_	*Lym*	502663	7896	195	95%	97%	5.00%
	*Den*	506484	4075	182	97%	98%	3.00%
	*Dip*	510727	768	192	99%	98%	1.40%
RF_V SURF_	*Lym*	502663	7896	29	97%	94%	5.60%
	*Den*	506484	4075	14	99%	96%	3.60%
	*Dip*	510727	768	8	99%	96%	3.60%

*The number of observations of non-defoliated FC (n_*y*_ = 0) and defoliated FC (n_*y*_ = 1) for the particular PPI may vary due to the occurrence (and exclusion) of missing values depending on the variable selection. The number of variables processed (n_*var*_) varied between RF approaches. The particular model accuracy is shown by the true positive rate (TPR), the true negative rate (TNR), and the out-of-bag error (OOB)*.

In the case of RF_clim_, observations of non-defoliated FC (n_y__=__0_) were limited to FC that experienced defoliation by the particular PPI at least once within the observation period, whereas the other RF approaches considered observations of all FC. Thus, RF_clim_ referred to a different data set focusing on the temporal occurrence of defoliation in relation to the respective climatic conditions rather than on the spatial distribution of the defoliation events. The statistical results were not directly comparable with the other RF approaches. Furthermore, RF_clim_ used by far the highest number of variables. This first step of analysis has been performed to (a) preselect the most important time windows and climatic drivers (Supplementary Table 1), and (b) to identify the best explaining structural variables based on different weightings and spatial aggregations of forest inventory data (Supplementary Tables 3, 4).

The 10-fold cross-validation process obtained a generally high performance of the applied RF classification. The last column of Table 1 shows the OOB calculated within the training procedure of the RF, whereas TPR and TNR of the classification resulted from the prediction on the entire forest area in Brandenburg. The poorest results were obtained for classifications based on stand variables exceeding an OOB of 10%. Except for RF_clim_ focusing on the temporal occurrence of defoliation events and a reduced set of observations (see above), the lowest error rates were observed for the combined variable set excluding highly correlated variables within one group (RF_all_). Both TPR and TNR showed the best results for *Dip* with 99% and 98% and the poorest results for *Lym* with 95% and 97%, respectively.

**FIGURE 5:**
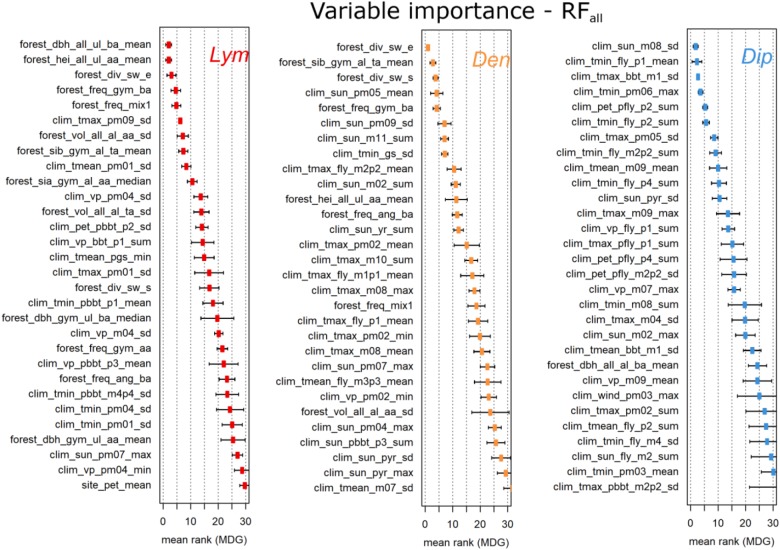
Top 30 ranks of the RF_all_ variables based on MDG. The three figures show the arithmetic mean and the standard deviation over the individual rankings within the 10-fold cross-validation process. Note that the data set of RF_all_ was cleansed of variables of low importance or high correlation (see the Section “Variable Importance”).

The variable importance of the first four RF approaches was determined by the arithmetic mean of the variable ranks based on the MDG measures of the 10-fold cross-validation process. To better illustrate the most important variables, we present only the top-30 ranked variables of RF_all_ (Figure 5). Similar illustrations showing the results for the different variable groups including highly correlated variables can be found in the Supplementary Figures 1–3.

The most important variables of the three PPI originated from the variable groups clim_ and forest_ (Figure 5). For *Dip*, 29 of 30 variables originated from clim_. In contrast, the top ranked variables for *Lym* and *Den* originated from the forest_ group followed by clim_ variables. The stand_ variables never occurred within the top 30. One site_ variable, the mean long-term potential evapotranspiration per FC (site_pet_mean), appeared at rank 30 in the results for *Lym*. For all three PPI the standard deviation of the ranking increased with lower ranks.

According to Figure 5, the top three variables of *Lym* were expressed by the mean diameter and height of all forest stands within 1,000 ha exclusively calculated for the upper layer of the forest stands, and the Shannon index calculated by the relative tree species composition. For *Den*, the top three were the Shannon index, the relative site factor of gymnosperms, and the standardized Shannon index within 1,000 ha. In the case of *Dip* the three most important factors were the standard deviation of the sun duration in August, the average minimum temperature of the first week of the flight period, and the standard deviation of daily maximum temperatures 1 week before BBT of pine.

For a fully automatic variable selection, we additionally applied the VSURF algorithm (see above, Genuer et al., 2015). The final models of this approach (RF_V SURF_) contained 29 variables suitable for predictions of *Lym* classification, 14 variables for *Den*, and 8 variables for *Dip* (Table 2).

**Table 2:** Variable selection of the three PPI based on the VSURF algorithm (RF_V SURF_) and variable ranks according to the MDA importance measure.

Rank	*Lym*	*Den*	*Dip*
1	site_ycoo	site_ycoo	clim_tmax_ pm05_sd
2	forest_hei_all_ul _ba_median	clim_tmax_fly _m3p3_sum	clim_ sun_m08_sd
3	forest_hei_all_ul _ba_mean	clim_sun_yr_sd	clim_sun _m02_max
4	clim_tmax_pm09_sd	clim_sun_pm05 _mean	site_ycoo
5	clim_pet_pbbt _p3_sd	clim_sun _pm07_max	clim_ vp_m07_max
6	clim_vp_ pm04_sd	clim_sun_pm04_max	clim_tmin_fly _m3p3_mean
7	clim_vp_bbt_p1_sum	clim_sun_pyr_sd	clim_ tmin_m09_sum
8	forest_freq_mix1	forest_div_sw_i	clim_tmin _pm06_max
9	clim_sun_ pm07_max	clim_sun_pm04_sum	
10	clim_tmax_pm01_sd	clim_sun_pm04_mean	
11	clim_sun_m04_max	clim_sun_pgs_max	
12	clim_vp_m04_sd	clim_sun_m06_max	
13	clim_tmean_pm01_sd	site_xcoo	
14	clim_vp_pm02_sd	clim_sun_m02_mean	
15	clim_tmean_pgs_min		
16	clim_sun_gs_sd		
17	clim_rain_bbt_m1_mean		
18	forest_dbh_all _al_aa_mean		
19	clim_rain_m10_sd		
20	clim_sun_pgs_sd		
21	clim_tmin_pm04_sd		
22	clim_vp_pfly_m1p1_sd		
23	clim_sun_pyr_sd		
24	clim_sun_m04_sd		
25	clim_sun_m09_mean		
26	site_pet_mean		
27	clim_sun_m09_sd		
28	clim_vp_pbbt_p2_mean		
29	clim_rain_m02_max		

The inclusion of the *x*–*y*-coordinates of the individual FC into our analyses exhibited a high predictive power of the latitude (see Figure 1B) and, respectively, a high variable importance (Table 2). Thus, the coordinates seem to be important variables for prediction purposes and, in turn, for the variable selection process of RF_V SURF_. In contrast to variable importance based on MDG (Figure 5), the VSURF algorithms predominantly selected variables from clim_ for all PPI. Furthermore, there is an obvious shift in the ranking of (VSURF-) MDA variable importance compared to the MDG measure of RF_all_ (e.g., for *Dip*, clim_tmax_pm05_sd switched from seventh to first rank; see Figure 5 and Table 2).

The analyses produced the simplest RF_V SURF_ model for *Dip*. The selected climatic variables are predominantly expressed by standard deviation (_sd) or maximum measures (_max). Almost twice the number of variables entered the RF_V SURF_ model for *Den*. The Shannon index was the sole variable of the forest_ variable group. The sum of the maximum temperature 3 weeks before and 3 weeks after flight date appeared as second-ranked variable for *Den*. All other variables originated from the group derived from the number of sun hours. Standard deviations of sun_ within the current year appeared at third rank, whereas the following ranks were taken up by climate aggregates of the previous year. Similar to *Lym*, the *y*-coordinate was the most important variable predicting defoliation of *Den*. The next important variables for *Lym* were the arithmetic mean and the median of the height of the upper layer of all forest stands within 1,000 ha. The first three climate variables of *Lym* expressed the standard deviation of previous-year climate data. The climate conditions of the current year were represented by the sum of the vapor pressure of the first week after BBT. Another important forest_ variable was the frequency of pure (monospecific) stands within 1,000 ha.

In order to highlight the most influential climate variables for the three PPI we compared the top ranked variables achieved for prediction (RF_V SURF_) and interpretation (RF_clim_). The latter approach considered only climate data of FC that had experienced at least one defoliation event within the last 15 years. We thus suppose that the RF_clim_ variable importance was predominantly driven by variables contrasting the individual years rather than reflecting the spatial distribution of the FC.

**FIGURE 6:**
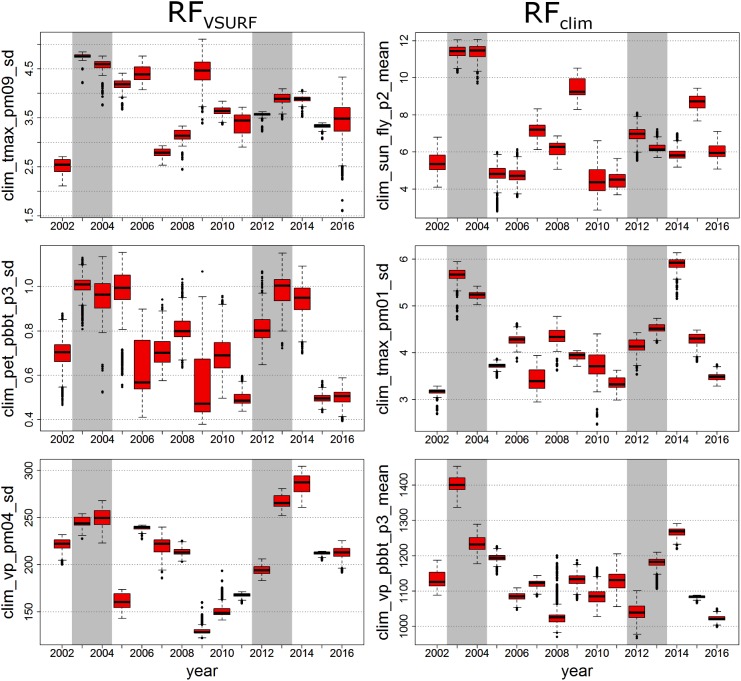
Boxplot representation of the annual distribution of the top three climate variables for *Lym* as suggested by RF_V SURF_ **(Left)** and RF_clim_ **(Right)**. Mass outbreak years are indicated by gray background coloring.

As an example for this approach, Figure 6 shows an annual boxplot representation of the three most important climate variables of *Lym* (see Table 2 and Supplementary Figure 1). Similar figures for *Den* and *Dip* can be found in the Supplementary Figures 4, 5. Note that the supplementary figures of variable ranking contain highly correlated variables, which were not considered for the selection of the top-ranked variables (see the Section “Variable Importance”).

Except for the first ranked RF_clim_ variable, the most important climate variables of *Lym* referred to previous-year climatic conditions (Supplementary Figure 1). RF_V SURF_ variables were expressed by the standard deviation (Table 2), whereas RF_clim_ showed measures of the arithmetic mean in the first and third ranked variables. In mass-outbreak years (gray bars), the selected variables frequently showed higher values compared to the mean level of all years (Figure 6). The outbreak years are marked by high mean sun duration in the first 2 weeks of the flight period of the actual year (RF_clim_ first variable) and high mean vapor pressure in the first 3 weeks after BBT in the previous year (RF_clim_ third variable). The second variable represents a high standard deviation of the maximum temperature of previous January. In RF_V SURF_, the top three variables of *Lym* outbreak years are characterized by a high standard deviation of maximum temperature of previous September, potential evapotranspiration 3 weeks after BBT of the previous year, and vapor pressure in previous April.

Summarizing the results of *Den* and *Dip* (Supplementary Figures 4, 5), we obtained a much clearer separation of mass outbreak years from the other years for the top three climate variables for *Dip* than for *Den*. In RF_V SURF_, the top three variables of *Den* outbreak years were related to a high (maximum) temperature sum 3 weeks before and after FLY, a low deviation of the sun duration in the previous year, and a low mean of the sun duration in May of the previous year. In RF_clim_, the top three variables are characterized by high means of the maximum temperature in the first week of the flight period and of the vapor pressure 3 weeks before and 3 weeks after previous FLY, and by a low maximum of the daily sun duration in previous July. In RF_V SURF_, the top three variables of *Dip* outbreak years showed a low standard deviation of the maximum temperature of previous May, a high standard deviation of the sun duration in August, and a low maximum of the sun duration in February. In RF_clim_, the top three variables are related to a high sum of the potential evapotranspiration in the 2 weeks after previous FLY, a low maximum of the minimum temperature of previous June, and a low sum of the minimum temperature in the first week after FLY.

**FIGURE 7:**
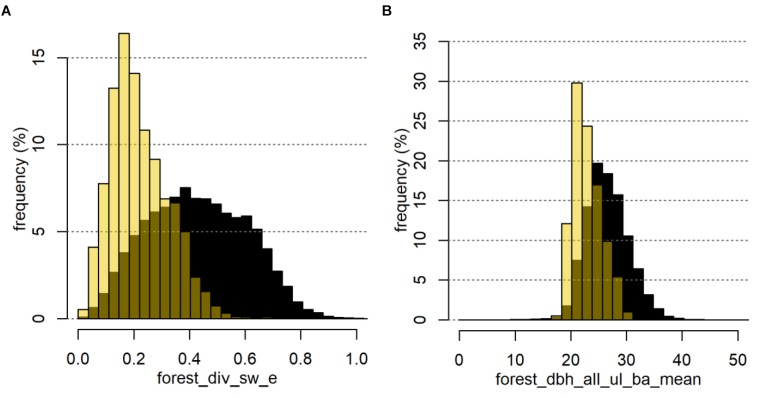
Example of two important forest_ variables for *Den* represented by the density distribution of the mean Shannon evenness (A) and the mean basal-area weighted stand diameter at breast height of all upper forest layers within the 1,000 ha buffer zone (B). Black bars show the distributions for all FC in Brandenburg, yellow bars show the distributions for defoliated FC only.

The top ranked forest_ variables allowed a clear differentiation of defoliated FC. In the example of *Den* (Figure 7), we observed a maximum value of the Shannon evenness of 0.6 for the defoliated FC (Figure 7A). The highest density of defoliated FC was reached for values from 0.16 to 0.18, whereas the distribution of Brandenburg showed highest densities of FC between 0.26 and 0.64. In accordance with a comparably low diversity and evenness, defoliated FC also showed a high proportion of gymnosperm forest stands with a minimum of 0.6. The most frequent mean-dbh classes in the defoliated FC were significantly lower than in the total of all FC (Figure 7B).

Similar relations could be found for *Lym* and *Dip* showing clearly different peaks for the respective variable classes of RF_forest_ (Supplementary Figures 6, 7). We obtained a comparable value range of the respective forest_ variables, e.g., for basal-area weighted mean diameter of the upper layer within 1,000 ha (Supplementary Figures 6A, 7A). For all PPI, defoliated FC were characterized by rather small tree dimensions associated with a stand age between 40 and 70 years, a low diversity as indicated by a low Shannon index or Shannon evenness, and a high proportion of gymnosperms, i.e., Scots pine stands.

**FIGURE 8:**
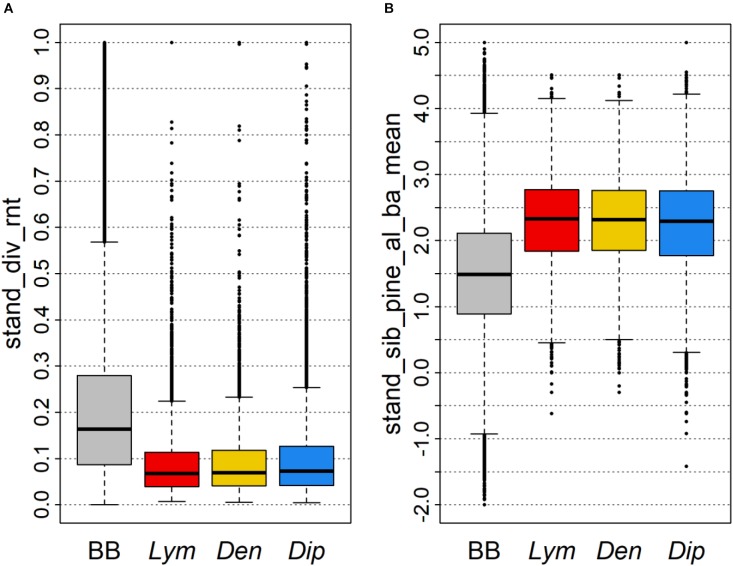
Distributions of two important stand_ variables shown as boxplots of **(A)** the relative number of tree species per hectare and **(B)** the mean relative site index of pine stands within a FC. The baseline shows all data (BB) whereas PPI boxplots were calculated for FC defoliated by *Lym, Den*, and *Dip*, respectively.

Although no stand_ variables were observed among the most important variables for classification of PPI mass outbreaks (Figure 5), a comparable differentiation of the defoliated FC from the distribution of all FC in Brandenburg was registered. As an example the mean number of tree species per hectare was lower (Figure 8A) and mean relative site index of pine stands was higher (Figure 8B) for PPI defoliated FC compared to the total of all FC in the state.

## Discussion

### Random Forest Variable Selection

We obtained a great performance of the RF algorithms handling huge databases of different PPIs. The settings for the RF and the applied methods proved to have a high accuracy in classification (Table 1). Even the sharply reduced sets of predictor variables selected by the VSURF algorithm (RF_V SURF_) obtained remarkably high rates of correct prediction (TNR) for more than 90% of the forest compartments defoliated by the three investigated PPI species. TNR results comparable to RF_V SURF_ were found for RF_forest_ and RF_all_ using around 500 and 200 variables, respectively, whereas classification by RF_stand_ was somewhat poorer.

The achieved high model accuracy is a result of large amounts of information representing manifold causal relations, but also of the constitution of the analyzed databases. Due to the characteristic spatial distribution of the defoliation events in Brandenburg, the RF algorithms search for variables that optimally separate defoliation hotspots from other regions. We started our analysis with a set of climatic variables that consider varying time windows from two subsequent years, so various data patterns are provided to describe both the observation period of 15 years (Figure 2) and the spatial distribution (Figure 1B). Therefore, variable importance measures may identify a random data pattern rather than causal relationships to produce the most appropriate classification of the analyzed information.

Nevertheless, the extension of the analysis to a huge number of variables may firstly help to characterize past defoliation of mass outbreaks and is secondly essential for providing promising variable sets which describe reliable ecological relationships. Floating time windows, in particular, may provide hints on critical periods of the year that impact the development of mass outbreaks and feeding intensity (Möller et al., 2017). Furthermore, the identified climatic factors may impact the population dynamic on pest insects either directly (Netherer and Schopf, 2010) or via the physiological response of the host plant (Lindner et al., 2014). In addition, important climatic variables may represent both critical phases in the population dynamics of the species’ natural antagonists and physiologically important periods according to particular biological needs (Chaves et al., 2003). Archaux and Wolters (2006) described the general impact of summer drought on forest biodiversity with decreased ecosystem productivity and increased mortality. The response to these extreme conditions depends on mobility, reproductive rate, or resistance mechanisms of the respective species. The consequences of climate change for the mostly host-specific natural antagonists of *Den, Lym*, and *Dip* are still largely unknown.

A critical remark has to be made on data availability: During the analyzed time covering 15 years of spatially referenced data we observed only two or three mass outbreaks, respectively (Figure 2). Depending on the pest species, mass outbreaks covered 1–3 years of defoliation in up to 2,000 forest compartments per year (Figure 2). The population dynamics of most forest pest insects follows a more or less fixed pattern for mass propagation with wave-shaped climax phases covering several years (Schwenke, 1978; Baltensweiler and Rubli, 1999; Hlásny et al., 2016). For example, mass outbreak periods of *Lym* occurred about every 10 years in Brandenburg. In the later years of these gradation cycles, the effect of the specific climatic triggers might become blurred by the effects of the initial population size in the previous year, even more so if feeding intensity is neglected as an influencing factor. In our analysis, we considered all defoliation classes as dichotomous “on-off” cases and thus accessed the maximal number of observations available. An exclusive focus on total defoliation (needle loss > 90%) events or the observation of specific phases of the population dynamic might further promote the identification of factors that trigger the onset of an outbreak cycle. In fact, the highest impact of the climatic conditions should be found before or during the first (“progradation”) phase of mass outbreaks directly preceding a steep increase in population size. This narrowed approach, however, would be constrained by a severe reduction in data availability.

Data quality heavily depends on the origin of the particular parameters. The reliability of the regionalized environmental data (clim_ and site_) is limited by the uncertainties of the particular model application (Russ and Riek, 2011; Köhler et al., 2015). The core data used for stand_ and forest_ characterization, in contrast, originated from forest inventory data with a re-sampling rotation of 10 years that are corrected by annual harvest and mortality data provided by the local forest managers. Information from this background introduces an additional level of uncertainty due to the potential subjective errors and temporally incoherent observation standards.

Since this study aimed to provide the most important explanatory variables for PPI defoliation inherent in the database, we computed different metrics of spatial and temporal data subsets for each set of parameters. While clim_ variables primarily explain the temporal appearance of PPI mass outbreaks, stand_ and forest_ variables should have a high impact on the spatial distribution of defoliation hotspots. Top variables of both variable groups clearly differentiated the continuum of forests attacked by PPI and the forest population of the whole study area (Figures 7, 8). On the landscape level, however, forest_ variables have proved to be most influential for classification and substituted stand_ variables in both variable selection approaches RF_all_ and RF_V SURF_ (Figure 5 and Table 2).

We suggest that both compared variable importance measures (MDG and MDA) reliably select the variables that best represent the spatial distribution of PPI feeding events. The particular realizations of these variables deliver detailed information on the insects’ ecological niche and, in turn, on the most likely feeding hotspots. However, the importance of clim_ variables largely depends on the particular setting of the RF, and the MDA measure might be misleading in some cases. In fact, the selection of standard deviation and maximum or minimum values (Table 2) may lead to the best representation of the pattern within the examined database without plausible biological links to the population dynamics of PPI. We also suppose that the consideration of climate data from all forest stands in the RF setting (RF_all_ and RF_V SURF_) introduces additional noise to the temporal trend due to the spatial pattern of the climate data from specific years. Therefore, we suggest the application of RF_clim_ to interpret climatic triggers and crucial periods of the year. Despite of manifold uncertainties and shortcomings in the observation data and methodological details we are nevertheless confident about the quality of the primary sets of influencing factors for the three PPI. The selection of the climate variables, however, has to be evaluated by the years to come which will test the robustness of the respective parameters.

Finally, we were able to analyze a huge data set by different approaches using the RF algorithms implemented in “R” and obtained a reliable variable selection. We suggest that the quality of the variable importance measure might be restricted rather by the sample size and the timeframe available than by technical issues. Nevertheless, some methodological issues resulting from a high number of correlated variables (see Genuer et al., 2010) and strongly unbalanced data (see Chen et al., 2004) have to be considered. We recommend to spend more effort on database management providing continuous information on the predictive variables and to use the RF algorithms to detect changes in the data patterns as well as in the importance of ecological variables.

### Ecological Relevance of Identified Classification Variables

The results achieved by the RF analyses are closely consistent with the known species-specific gradation patterns and their changes in the recent past. If a species exhibits a strong temporal regularity in the frequency of mass outbreaks, a tight dependence between population dynamics and the stand conditions (which remain more or less constant over a long time) has to be assumed. Consequently, the irregularity of mass outbreak patterns increases with a rising importance of climatic variables. Our findings indeed revealed a strong dependency of *Dip* mass outbreaks on climatic drivers (Figure 5 and Table 2) which complies with the importance of the bivoltine lifecycle of *Dip* in Brandenburg and its close coupling to the climatic conditions of the current and the previous year (Möller et al., 2017). In contrast, *Lym* showed a higher variable importance for parameters of the forest_ variable group and a rather strong temporal regularity of outbreak events (Gräber et al., 2012).

Outbreak patterns and practical experiences have influenced the monitoring methods in forestry for a long time (e.g., Schwerdtfeger, 1934, 1981; Wellenstein, 1942; Sierpiñska, 1998; Häußler et al., 2000). The results of our investigations can help to further improve the forecast of timing and intensity of mass outbreaks and to adapt the respective methods to a changing environment. A reliable prognosis of the amplitude during the outbreak culmination, i.e., the infestation area and the intensity of needle loss to be expected, in dependence on the floating climatic windows could provide important essentials for evaluating and enhancing the current monitoring methods.

The obtained results on critical climatic factors correspond closely both with findings published by forest entomologists more than 100 years ago (e.g., Altum, 1881; Zwölfer, 1935) and with observations made by forest practitioners. A comparison of the found important climatic windows with the individual development of the species shows that there are a number of biologically plausible explanations for the statistical outcome. The three investigated pine pest species share a common preference for middle-aged, poorly structured pine forests. The successful development of mass gradations, however, depends on different critical climatic phases of the year relating to their specific life cycles.

#### Lymantria monacha

In accordance with other forest moths, it is widely accepted that *Lym* is highly sensitive toward temperature and prefers warm conditions. Vanhanen et al. (2007) suggested a northward shift of the range of *Lym* in the future triggered by warming climate conditions. Our study revealed some important climatic windows which strongly influence the development of this species.

The amplitude of an outbreak’s culmination in terms of feeding damage depends heavily on the conditions in the initial phase of the outbreak. Increased sunshine hours during September of the previous year (clim_tmax_pm09_sd) have a positive effect on the severity of feeding. Zwölfer (1935), for example, observed high mortality rates in *Lym* eggs if they cannot finish their embryonal development in autumn due to detrimental weather. The egg stage shows three phases. The first phase is the highly temperature-dependent embryonic development of 2 up to 6 weeks following oviposition. The second phase is a hereditary development dormancy lasting for another 10 weeks; the third and last phase is winter rest due to low temperatures (Schwenke, 1978). Our findings emphasize the importance of the second phase for the survival of the young larva hibernating inside the egg.

The dominant influence of temperature on egg and larval development is further confirmed by the high rank of variation in average temperatures in January of the previous year (clim_tmean_pm01_sd). Winter conditions determine an insect’s energy balance in a fundamental way. Temperature is an essential factor for egg and larvae metabolism even if other factors such as ambient moisture, nutrition status, or biotic opponents may also heavily influence dormancy regulation (Müller, 1992). A low mortality pressure on the parent generation caused by conditions favorable for the development during winter dormancy, for example, increases population density which in turn could lead to a mass outbreak in the next year (Wellenstein, 1942). The importance of vapor pressure in April of the previous year (clim_vp_pm04_sd) is related to the fact that intense drought periods can lead to raised egg mortality caused by desiccation (Wellenstein, 1942). This is one reason why egg vitality in winter should be investigated to further specify the forecast of feeding damage by *Lym*.

The effects of forest structure on the occurrence of mass outbreaks and defoliation were most significant for *Lym* as compared to *Den* and *Dip.* The largest and most severe *Lym* gradations commonly occur in pure conifer stands (Altum, 1881; Schwenke, 1978) with the highest proportion of defoliated pine stands located in forests with a dbh range of 20–24 cm for the dominant trees (Supplementary Figure 6). Similar results were found at the level of the individual forest compartments (stand_ variables) but are not shown here due to a lower explanatory power of this variable group compared to the forest_ variables. The high rank of the variable forest_div_sw_e, the standardized Shannon-Weaver index averaged over the 1,000-ha buffer area, proves that increasing tree species diversity reduces the risk of mass outbreaks. Experiences from forestry practice and from the monitoring system also show that *Lym* outbreaks often start in large areas covered by pure pine stands, which are inherently poor in structure and productivity (Figures 7A,B).

In Brandenburg, *Lym* has been characterized as the most important PPI responsible for widespreading defoliation of pine forests in the past (Gräber et al., 2012). Obviously, warm and dry conditions promote mass outbreaks of *Lym* as shown by the huge number of defoliated FC in 2003 (Figure 2). Once a mass gradation has successfully begun, however, high population densities seem to uncouple infestation from climate and severe defoliation can be expected in the following year. The large proportion of pure pine stands in Brandenburg represents favorable stand conditions for *Lym* supporting the observed temporal regularity of mass outbreaks. While stand structures alter with time and may be improved by forest management activities that increase the number of mixed and deciduous forest stands, climate change may again raise the biotic risk of defoliation. In addition, *Lym* might be quite adaptable to changes in the forest structure due to its polyphagous nature. Thus, future feeding events should be analyzed for changes in the feeding preferences of *Lym* and characterization of forest stands’ predisposition.

#### Dendrolimus pini

The pine-tree lappet moth’s feeding preferences for middle-aged pure pine stands are similar to those of the nun moth as shown by the variable ranking of the forest_ variables (Figures 5, 8B). In fact, mass outbreaks of *Den* frequently follow the observed outbreaks of *Lym* (Figure 2) in almost the same infestation areas (see Figure 1B, where orange-colored FC indicate *Den* and *Lim* outbreaks in identical FC with yellow representing *Den* and red color representing *Lim*). Thus, *Den* is a harmful forest pest in Brandenburg feeding on already weakened pine stands. Furthermore, the heavy defoliation of juvenile plants associated with this species may lead to the decline of the entire forest. Similar to *Lym*, the pine-tree lappet moth prefers warm conditions suggesting an increasing risk of defoliation in the future.

The duration of sunshine during May of the previous year (Figure 5: clim_sun_pm_05_mean), for instance, is highly relevant for the classification of *Den* feeding events. More sunshine is related to higher temperature and less precipitation. Under these conditions the feeding intensity of the poikilothermic caterpillars increases, and a better vitality of the larvae can be assumed. The pupae stage will be reached earlier and the probability that larval parasitoids find their host is reduced. Additionally, faster growth increases the possibility that eggs of *Tachinidae* will slip off during host molting before penetration (Herting, 1960). Some Tachinid species lay their eggs on pine needles and after hatching the egg larvae must “wait” for host caterpillars. It can also be expected that hot and dry conditions shorten the life span of the parasitoids’ eggs and especially the egg larvae. In consequence, a sunny May promotes both a high vitality of *Den* and a reduced biological control by parasitoids.

Warm and dry conditions in late summer during the flight, copulation, and egg deposition phase as reflected by the variable clim_tmax_fly_m2p2_mean (Figure 5) further promote a high population density of *Den* (Weckwerth, 1952; Majunke, 2000; Ray et al., 2016). Sunshine during September (clim_sun_pm_09_sd) is positive for the development of the thermophilic egg larvae that represent the most sensitive development stage of the insect’s life cycle. Because the development of *Den* starts in one summer and ends after overwintering as caterpillar in the next one, it is biologically plausible that data of the previous year are statistically important for classifying population dynamics.

The effect of maximal temperature in October (Figure 5: clim_tmax_m10_sum) is also coherent with individual development requirements: During the last feeding period before hibernation the feeding intensity of the caterpillars is predominantly controlled by temperature. The amount of energy reserves deposited as storage proteins and lipid bodies (Levenbook, 1985) determines individual fitness (Kätzel and Möller, 1993) and is crucially important for survival during winter and the power to climb up the tree in spring.

The identification of sunshine duration in February (Figure 5: clim_sun_m02-mean) as a significant climatic window is quite interesting. The caterpillars of *Den* hibernate as L3 or L4 in the soil layer, and soil temperature determines the start of their climbing up the tree at the end of winter. At 1°C the caterpillars start to leave the soil, while at 6°C this process is culminating (Schwenke, 1978). In pine stands, the influence of sunshine on soil temperature is high because reflection and diffusion of incoming radiation by the crowns are comparably weak (Larcher, 1987). Leaving the soil earlier under favorable climatic conditions can significantly increase the survival rate for caterpillars because their overwintering in the soil is associated with numerous risks. These comprise natural enemies such as wild boar, mice, and entomophagous fungi, but also soil moisture promoting mortality by bacteria or fungi. Altum (1881) wrote that warm southeastern winds and low air humidity promote the process of ending hibernation. This knowledge is used in forest protection practice to start monitoring the climbing animals by estimating the number of larvae per crown at the right moment. This approach has proved to be an effective method to forecast *Den* feeding risks.

#### Diprion pini

In Brandenburg, mass outbreaks of *Dip* including widespreading defoliation events in the past were coupled to a bivoltine life cycle and second generation larvae feeding on needles (Möller et al., 2017). Since a successful development of two generations within 1 year depends on different critical phases for the respective development stages, mass outbreaks were predominatly controlled by climatic drivers. In contrast to *Lym* and *Den*, massive defoliation by *Dip* could almost exclusively be explained by climate variables (Figure 5 and Table 2). We also observed a slightly higher variance of the forest_ parameters (e.g., the mean diameter of the upper stand layer) for FC defoliated by *Dip* in comparison to *Lym* and *Den* (Figure 7B and Supplementrary Figures 6, 7). We thus suggest that if the climatic conditions favor a change to bivoltine reproduction, the quality of the host needles might be less important and *Dip* may affect a broader range of forest structures.

The monophaguous *Dip* is restricted to pine forests and population density is influenced by the quality of the feeding and breeding ground, even if forest_ and stand_ variables in our study have only minor explanatory power. For example, and also based on RF methods, Blomqvist et al. (2016) found lichen and lingonberry coverages to be the best predictors for cocoon mortality of *Dip* in Finland. Coverage densities were negatively correlated with mean defoliation intensity. Unfortunately, our data did not include detailed information on the herb layer. We nevertheless have to assume that the sensitivity to climatic drivers and the dependency on optimal forest structures in Finland differ from those in our study area because the life cycle of *Dip* in Finland even in outbreak years is predominantly univoltine (Geri, 1988). In northeast Germany these years are related to bivoltine phases, a situation that occurs rarely and is difficult to predict (Möller et al., 2017).

The prediction of this change from univoltine to bivoltine reproduction cycles might be the key to more reliable risk assessments for *Dip* mass outbreaks and severe defoliation. Shifts of the life cycle are probably related to a changing hierarchy of influential factors which should be considered in both model development and application. Flexible, phenology-oriented climatic “windows” can support the prognoses of critical development stages of *Dip* and of the risk of second generation feeding. In a previous study, we showed that recent mass outbreak events of *Dip* in Brandenburg were related to comparably low temperatures during the flight season, scarce precipitation in the period of BBT, a low variance of the mean temperature of the previous-year growing season and high evapotranspiration during the flight period of the previous year (Möller et al., 2017). Except for the precipitation variable, our results correspond to these findings (Supplementary Table 1). Deviating from the data set used in Möller et al. (2017), our analyses considered the complete forest area of Brandenburg. This might explain why precipitation data with their naturally high temporal and spatial variability were less influential here.

As a current example, the massive outbreak in 2016 in southern Brandenburg demonstrated the high risk potential of *Dip* if favorable climatic conditions are met. Therefore, we need to further investigate those climatic phases that are of crucial importance for the shift into the bivoltine life cycle of *Dip*. The results should be applied to the existing monitoring programs supplementing the risk assessment by count data.

#### Forest Protection

Many findings presented for the individual species are important arguments for management strategies that increase tree-species richness in forests. More and wider differentiated habitats for phytophagous species lead to better conditions for predatory zoophagous species and support an increased diversity in parasitoids as natural antagonists of insect pests like *Lym, Den*, and *Dip* (Kratochwil and Schwabe, 2001). A range of studies show the positive effect of deciduous trees in conversed pine forests as detectable in lower infestation by phytophagous insect pests (e.g., Hunter, 2001; Schulz and Dreger, 2003; Jäkel and Roth, 2004). As an example, Rös et al. (2004) found that population densities of the pine beauty moth *Panolis flammea* were strongly influenced by the proximity of deciduous forests, presumably due to the benefits for parasitoids such as tachinids.

In addition to the importance of climatic triggers of PPI outbreaks, we could show that the quality of the feeding ground and the structural forest properties clearly determine the spatial distribution of PPI feeding events. We successfully implemented the large body of information provided by forest inventories into our analyses and derived detailed risk assessments on the level of the individual forest compartment. Thus, we want to emphasize the importance of continuous forest inventory programs and the required collaboration of forest management and forest protection services.

The large areas of poorly structured pine stands of young to middle age, in particular in the South of Brandenburg, provide optimal conditions for PPI. In fact, common preferences of the individual PPI for forests with comparable stand structures may lead to parallel and/or sequential feeding in subsequent years which further elevates the risk for devastating defoliation. We thus need to forecast the development of these stands by forest growth simulations if we want to predict future feeding hotspots. Moreover, we observed a stronger predictive influence of forest properties of the neighborhood compared to the properties of the individual stands. Therefore, the predisposition of a FC toward the biotic risk of PPI may be altered by improving the structural features of the surrounding forest area, e.g., by increasing the proportion of mixed stands. Consequently, a higher number of tree species within one FC (Figure 8A) is linked to a reduction of the share of pure pine stands in the total forest area.

In agreement with findings from literature, pine stands at less productive sites as indicated by a higher relative site index have been most prone to defoliation by PPI (Figure 8B). Unfortunately, these stands are less suited for forest conversion and active enhancement of forest structural and species diversity due to their limited nutrient and water supply. The significant impact of the conditions in a larger buffer area, however, may help to promote individual FC by forest conversion activities at landscape level decreasing the overall predisposition for PPI feeding.

## Conclusion

This study provides a novel method analyzing a huge set of environmental data with regard to their influence on mass outbreak and defoliation events of PPIs. We have outlined the most promising environmental parameters for modeling the risk of mass outbreaks of *Lym, Den*, and *Dip* in Brandenburg. Hence, other research groups might benefit from our findings in the variable selection and modeling processes when performing risk assessments of PPIs on independent data sets. Our analysis showed both the high impact of the climatic conditions in particular for pest species with irregular mass outbreak patterns (e.g., *Dip*) and the importance of stand structures for the predisposition to defoliation by insects with relatively constant outbreak cycles (e.g., *Lym*). Furthermore, we could demonstrate a successful combination of detailed forest inventory data for large areas, regionalized climatic conditions, and site properties supporting forest protection issues and prognosis of future risk levels. The increasing availability of geo-referenced forest data in combination with novel data mining techniques can enhance our knowledge of forest pest insects’ population dynamics on landscape level and risk management, respectively.

We showed that (a) the PPI are sensitive to independent climatic triggers and temporal periods within the year relating to their species specific biology. The investigated PPI exhibited preferences to particular stand structures and specific adaptations to ecological (sub-) niches of pine forest ecosystems. However, mass outbreaks of all three PPI concentrated on low structured, young to mid-aged pine forests on rather poor sites. Therefore, we could not fully validate hypothesis (b) because there was no clear differentiation between the general forest types required by the individual species for successful mass outbreaks; all three insect species obviously prefer pure pine stands of lower dimensions. Furthermore, we demonstrated that PPI share (c) a common preference for warm climate and that (d) a higher tree species diversity of the habitat is counteracting severe mass outbreaks due to the limiting effects exerted by the higher abundance of predators and parasitoids under these conditions.

## Author Contributions

RH had the original idea for this study and covered the statistical methodology and data bank structuring and analyses. KM provided background knowledge on species biology and population dynamics. AW contributed essential monitoring and ecological data. AD was responsible for forest structure and stand variable integration. JS took care for combining all parts into the final manuscript, for the logical structure of the paper and for language and terminology issues.

## Conflict of Interest Statement

The authors declare that the research was conducted in the absence of any commercial or financial relationships that could be construed as a potential conflict of interest.
