# Supplementary figures and images for: Importance of Ecological Variables in Explaining Population Dynamics of Three Important Pine Pest Insects
Source: Front Plant Sci. 2018 Nov 13;9:1667. doi: 10.3389/fpls.2018.01667 (PMC6243470; doi:10.3389/fpls.2018.01667)

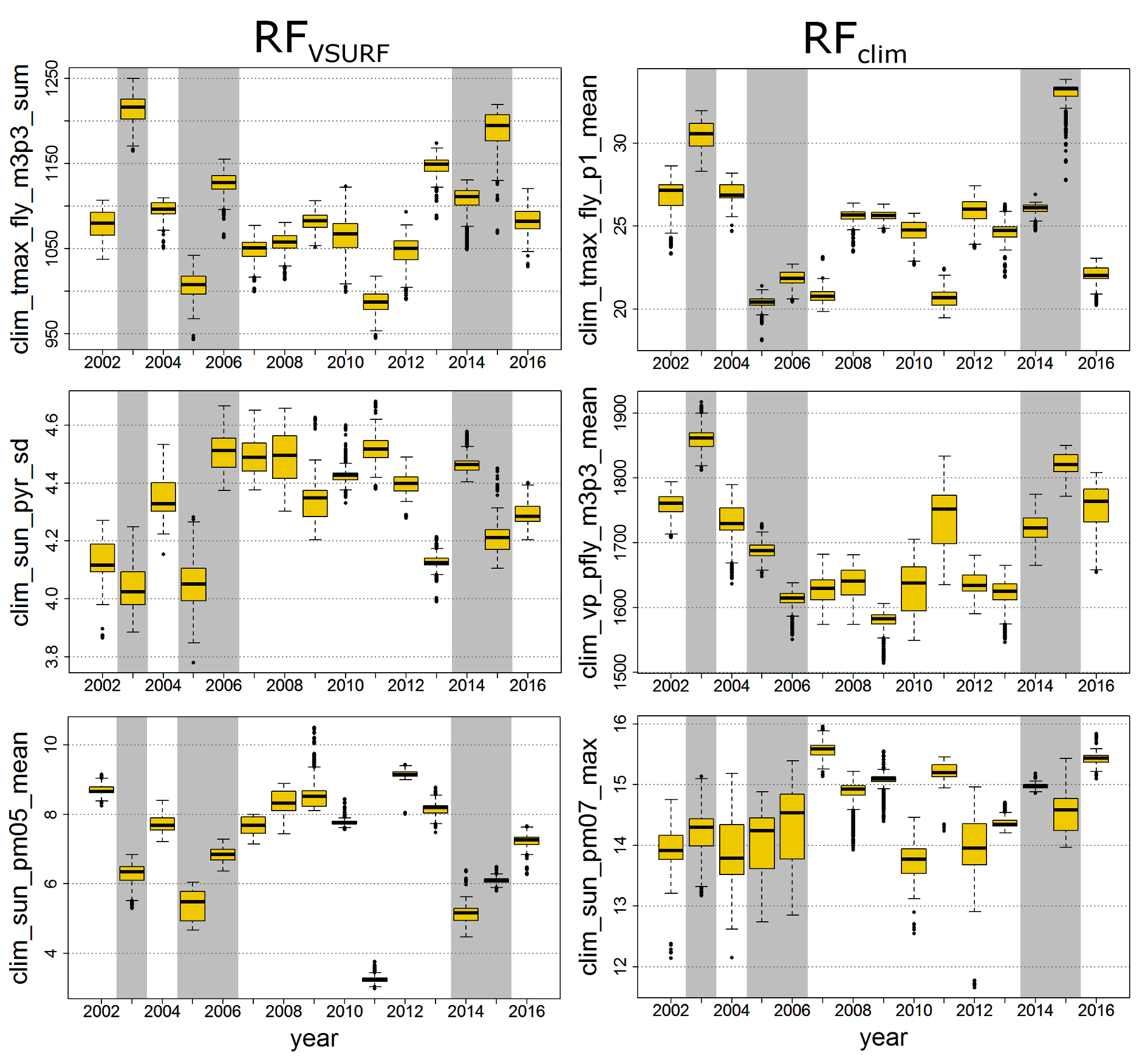

Supplement: Supplementary file 4 [file Image_4.TIF]

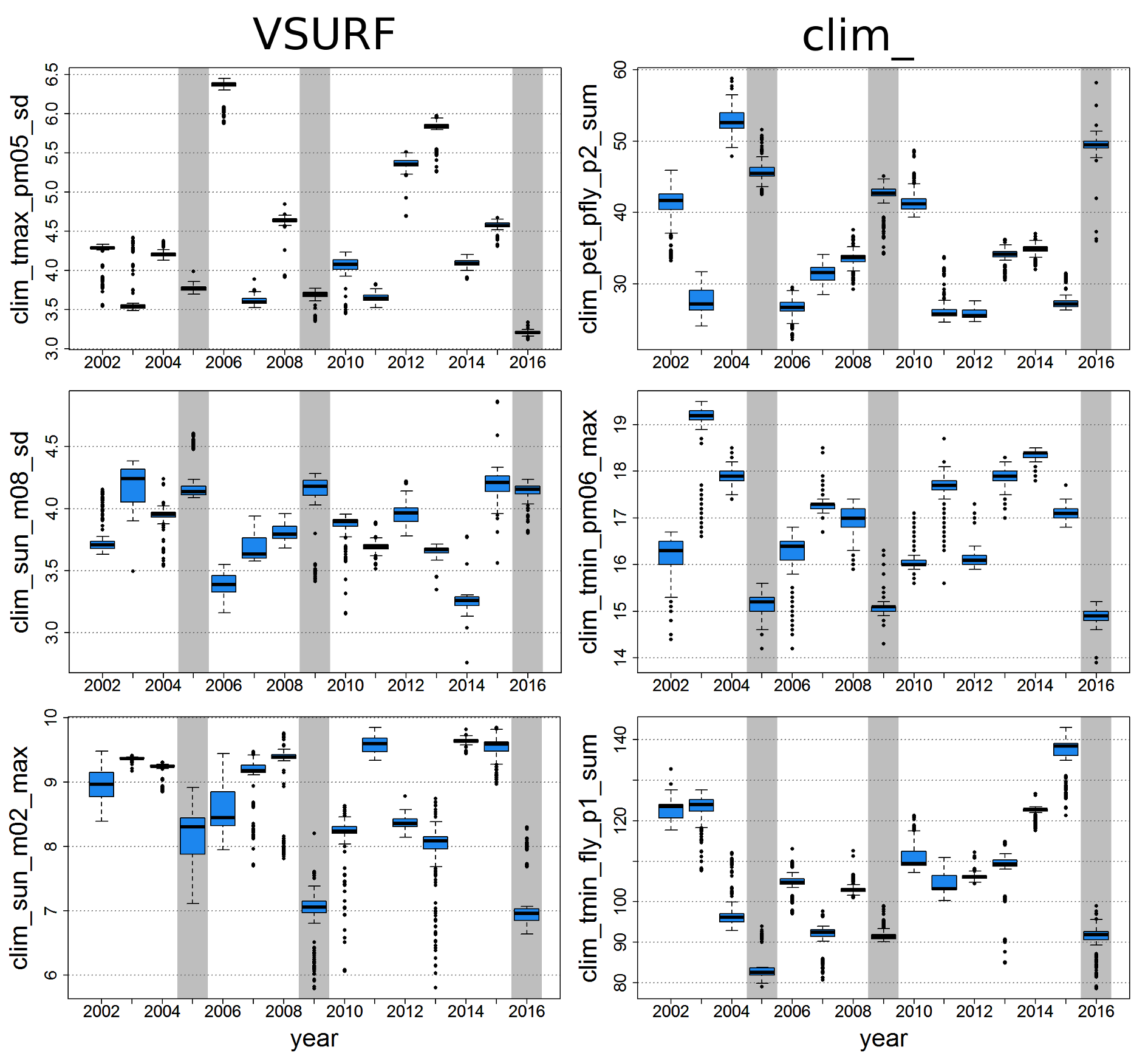

Supplement: Supplementary file 5 [file Image_5.TIF]

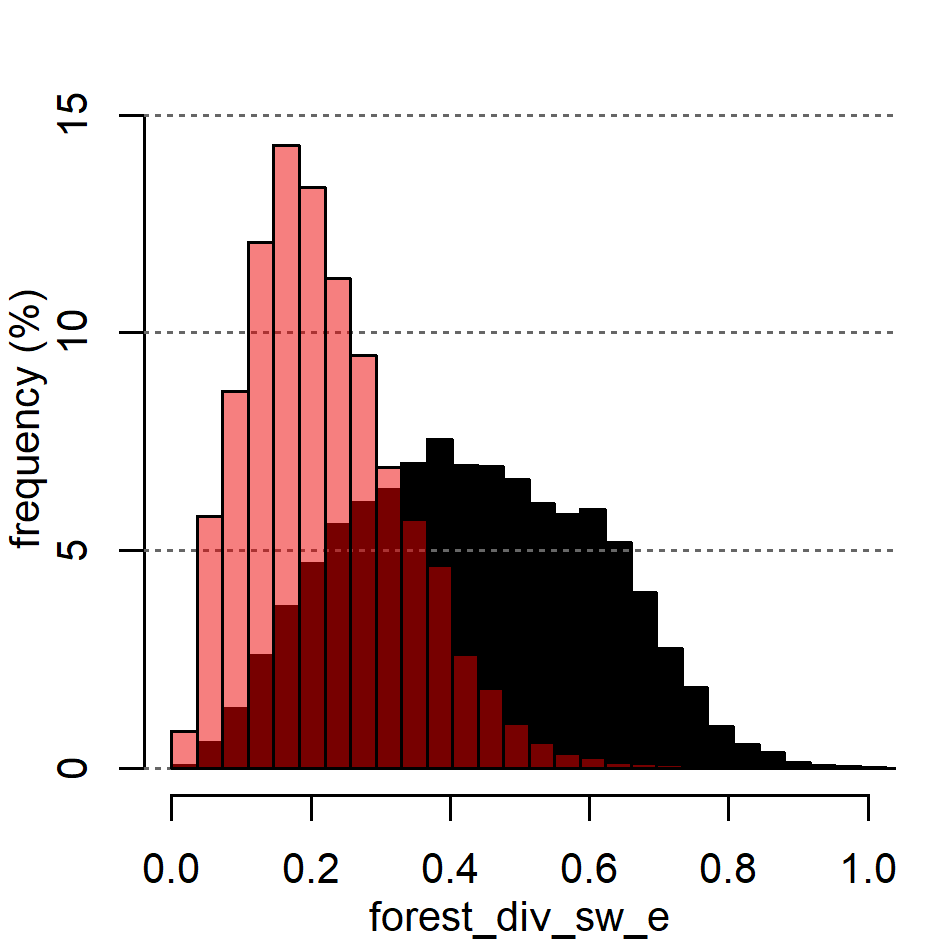

Supplement: Supplementary file 6 [file Image_6.TIF]

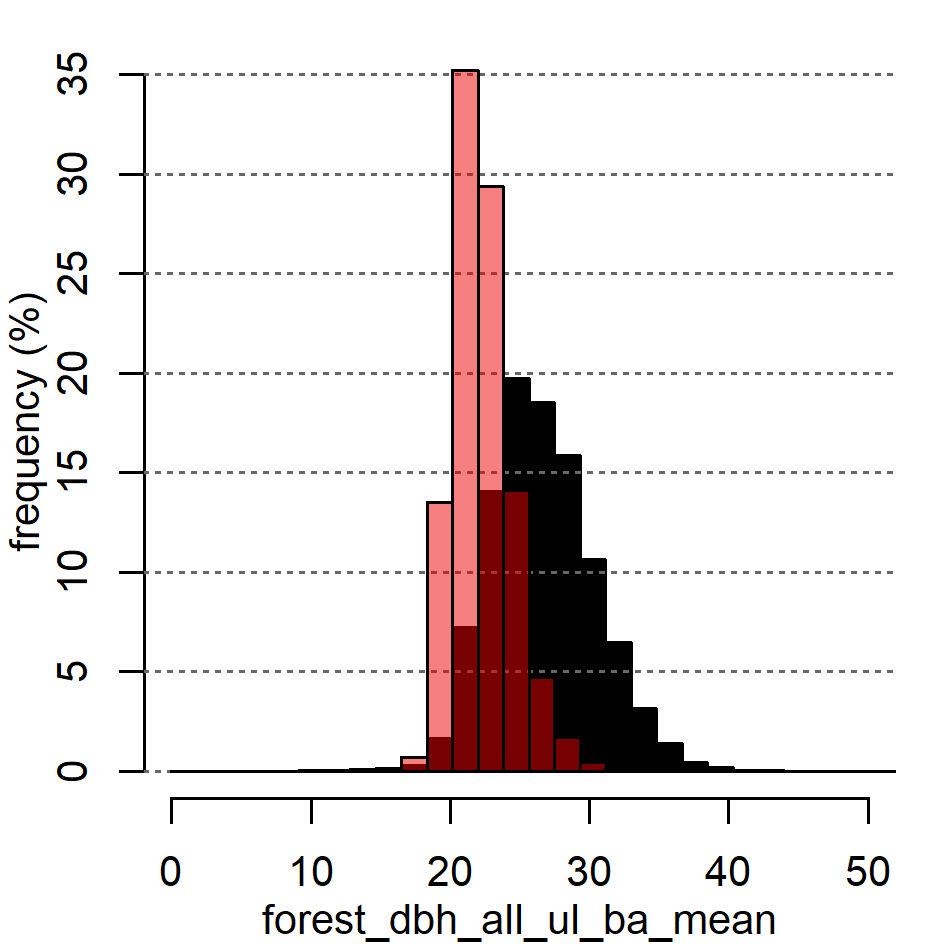

Supplement: Supplementary file 7 [file Image_7.TIF]

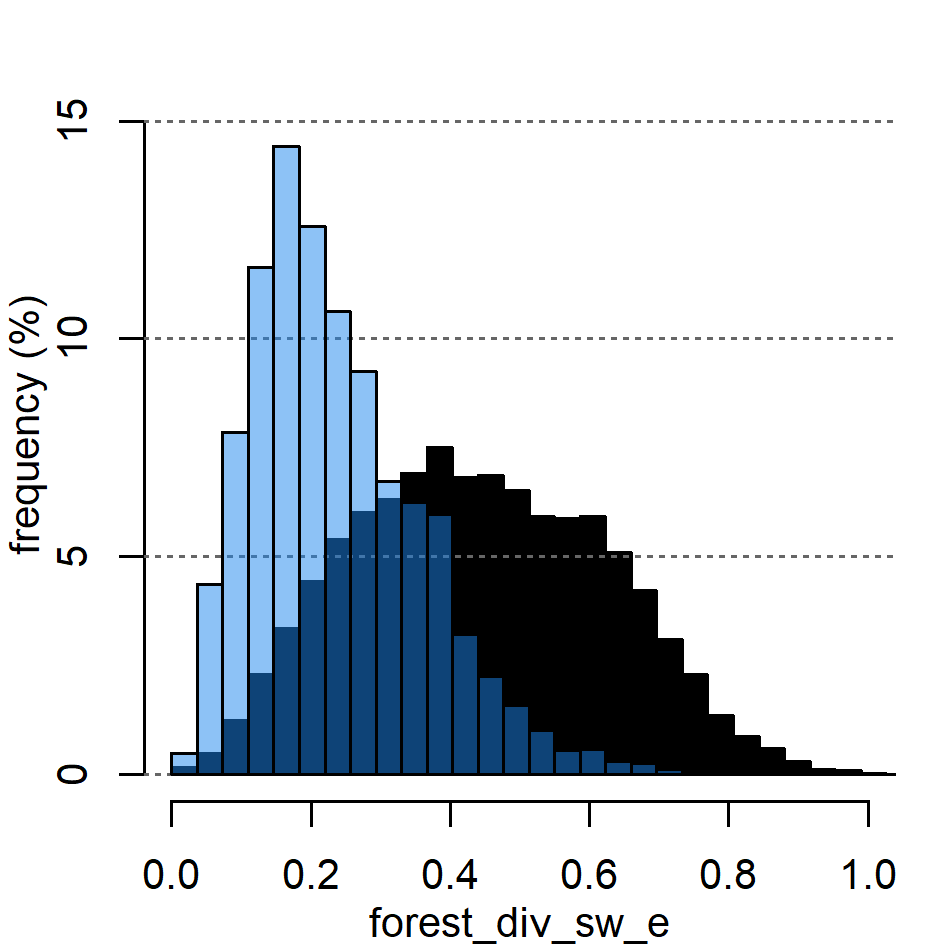

Supplement: Supplementary file 8 [file Image_8.TIF]

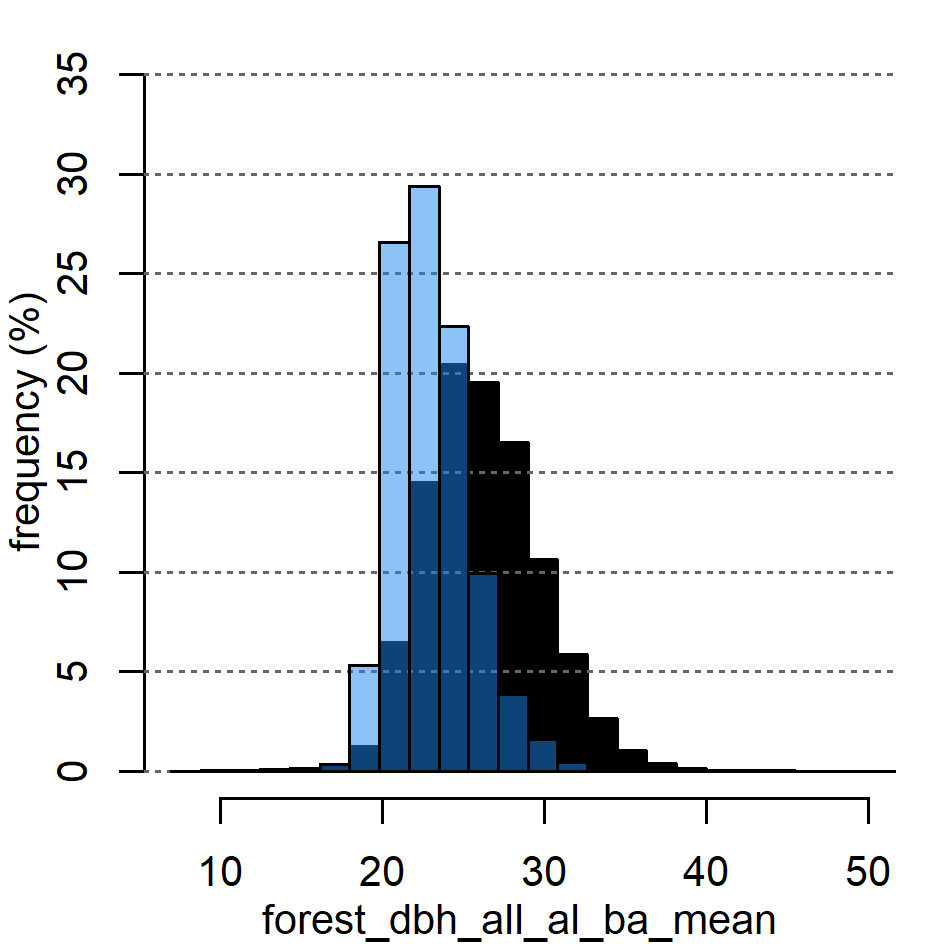

Supplement: Supplementary file 9 [file Image_9.TIF]
