# Supplementary material for: Importance of Ecological Variables in Explaining Population Dynamics of Three Important Pine Pest Insects
Source: Front Plant Sci. 2018 Nov 13;9:1667. doi: 10.3389/fpls.2018.01667 (PMC6243470; doi:10.3389/fpls.2018.01667)
Supplement: Supplementary file 10 [file Table_1.DOCX]

**Supplementary Table 1:** Description and encoding of the climatic variables. The acronyms of the individual variables were built by adding the first column entries to the respective position (starting with clim_ at 1^st^ position) in the code string. The climate parameters (2^nd^ position) represent daily values per FC as determined by the regionalization process of climate measurements.

| **Parameter** (2^nd^ position) | | **Description** | | |
| --- | --- | --- | --- | --- |
| tmin_ | Minimum temperature (°C) | | | |
| tmean_ | Average temperature (°C) | | | |
| tmax_ | Maximum temperature (°C) | | | |
| pet_ | Potential evapotranspiration (mm) | | | |
| rain_ | Precipitation (mm) | | | |
| sun_ | Sunshine duration (h) | | | |
| vp_ | Vapor pressure (Pa) | | | |
| wind_ | Wind speed (m s^-1^) | | | |
| **Temporal composite^1^** (3^rd^ position) | | | | **Description** |
| yr_ | Year | | | |
| gs_ | Growing season (01.04.-30.09.) | | | |
| m01…12_ | Month of the year | | | |
| bbt_ | Day of bud burst (modeled) | | | |
| fly_ | bbt plus 90 days | | | |
| **Temporal aggregate^2^** (4^th^ position) | | | **Description** | |
| m1…4_ | One up to four weeks before | | | |
| p1…4_ | One up to four week after | | | |
| m1…4p1…4_ | One up to four weeks before and after | | | |
| **Value** (5^th^ position) | **Description** | | | |
| mean | Arithmetic mean | | | |
| sd | Standard deviation | | | |
| sum | sum | | | |
| ^1^ “p” has to be added as first letter of the acronym if data originate from the previous year | | | | |
| ^2^ available only for bbt_ and fly_ | | | | |
