# Supplementary material for: Importance of Ecological Variables in Explaining Population Dynamics of Three Important Pine Pest Insects
Source: Front Plant Sci. 2018 Nov 13;9:1667. doi: 10.3389/fpls.2018.01667 (PMC6243470; doi:10.3389/fpls.2018.01667)
Supplement: Supplementary file 11 [file Table_2.DOCX]

**Supplementary Table 2:** Description and encoding of the site properties. The acronyms of the individual variables were built by adding the first column entries to the respective position (starting with soil_ at 1st position) in the code string. The soil parameters (2^nd^ position) represent basic soil properties per FC as determined by the regionalization process of edaphic measurements and the geographic location of the FC.

| **Parameter** (2^nd^ position) | **Description** |
| --- | --- |
| afc_ | Actual field capacity (mm) |
| asw_ | Plant available soil water (mm) |
| pet_ | Long-term potential evapotranspiration (mm) |
| aet_ | Long-term actual evapotranspiration (mm) |
| nv_ | Numerical nutrient value (-) |
| xcoo | Longitude of the center point of the FC (dd) |
| ycoo | Latitude of the center point of the FC (dd) |
| **Value** (3^rd^ position) | **Description** |
| mean | Arithmetic mean |
| sd | Standard deviation |
