# Supplementary material for: Importance of Ecological Variables in Explaining Population Dynamics of Three Important Pine Pest Insects
Source: Front Plant Sci. 2018 Nov 13;9:1667. doi: 10.3389/fpls.2018.01667 (PMC6243470; doi:10.3389/fpls.2018.01667)
Supplement: Supplementary file 12 [file Table_3.DOCX]

**Supplementary Table 3:** Description and encoding of the stand description part 1. The acronyms of the individual variables were built by adding the first column entries to the respective position (starting with stand_ or forest_ at 1^st^ position) in the code string. The stand parameters (2^nd^ position) represent measurements of the forest inventory partially complemented by regression models.

| **Parameter** (2^nd^ position) | | | **Description** |
| --- | --- | --- | --- |
| age_ | Age (year) | | |
| dbh_ | Mean diameter at breast height (cm) | | |
| hei_ | Mean height (m) | | |
| baa_ | Basal area (m^2^ ha^-1^) | | |
| vol_ | Wood volume (m^3^ ha^-1^) | | |
| sia_ | absolute site index (m) | | |
| sib_ | relative site index (-) | | |
| **Tree selection** (3^rd^ position) | | | **Description** |
| all_ | | All forest stands | |
| ang_ | | Angiosperm stands (= broadleaves) | |
| gym_ | | Gymnosperm stands (= conifers) | |
| pine_ | | Scots pine (*Pinus sylvestris*) stands | |
| oak_ | | Oak (*Quercus petraea* and *Qu. robur*) stands | |
| **Layer selection** (4^th^ position) | | | **Description** |
| al_ | | All forest layers | |
| ul_ | | Upper forest layers | |
| **Weighting** (5^th^ position) | | | **Description** |
| aa_ | | Absolute area of forest floor | |
| ba_ | | Basal area of resp. forest stands | |
| ta_ | | True area according to relative forest proportion | |
| **Value** (6^th^ position) | | | **Description** |
| mean | Arithmetic mean | | |
| sd | Standard deviation | | |
| median | Median | | |
