# Supplementary material for: Importance of Ecological Variables in Explaining Population Dynamics of Three Important Pine Pest Insects
Source: Front Plant Sci. 2018 Nov 13;9:1667. doi: 10.3389/fpls.2018.01667 (PMC6243470; doi:10.3389/fpls.2018.01667)
Supplement: Supplementary file 13 [file Table_4.DOCX]

**Supplementary Table 4:** Description and encoding of the stand description part 2. The acronyms of the individual variables were built by adding the first column entries to the respective position (starting with stand_ or forest_ at 1^st^ position) in the code string. Here, the parameters (2^nd^ position) were built for two groups of variables addressing the diversity and the relative proportion of specific stand classes.

| **Parameter** (2^nd^ position) | | | | | | | **Description** |
| --- | --- | --- | --- | --- | --- | --- | --- |
| div_ | | | | | | | Diversity measures |
| freq_ | | | | | | | Frequency |
| **Specification div_** (3^rd^ position) | | | | | **Description** | | |
| nf ^1^ | | | | | | | Number of forest types |
| nt ^1^ | | | | | | | Number of tree species |
| nt5 ^1^ | | | | Tree species with basal area >= 5% of total stand basal area | | | |
| sw | Shannon index (_i), standardized index (_s), maximal diversity (_m), evenness (_e) | | | | | | |
| si ^2^ | | Simpson index weighted by aa_ (_a), ta_ (_t), ba_ (_b) | | | | | |
| **Specification freq_** (3^rd^ position) | | | | | | **Description** | |
| [genus] | | | Proportion of forests of angio- and gymnosperm | | | | |
| [group] | | | Tree selection according to the Supplementary Table 3 (3^rd^ position) | | | | |
| age20…160 | | | Proportion of forests according to 20 year age-classes | | | | |
| mix1…6 | | | Proportion of forests from pure stands (_1) to mixed stands (_6) | | | | |
| lay1…3 | | | Proportion of forests of one (_1), two (_2) and more tree layers (_3) | | | | |
| reg | | | Proportion of forests with a regeneration layer | | | | |
| ^1^ “r” is added as first letter of the acronym for *relative* expressions (ha^-1^)  ^2^ weighting factors are described in Supplementary Table 3 (5^th^ position) | | | | | | | |
