# Supplementary material for: Importance of Ecological Variables in Explaining Population Dynamics of Three Important Pine Pest Insects
Source: Front Plant Sci. 2018 Nov 13;9:1667. doi: 10.3389/fpls.2018.01667 (PMC6243470; doi:10.3389/fpls.2018.01667)
Supplement: Supplementary file 14 [file Table_5.docx]

## Supplementary Figures – Captions

**Supplementary Figure 1.** Top 30 ranks of the RF_clim_ variables based on the MDG measure. The three figures show the arithmetic mean and the standard deviation of the ranking within the 10-fold cross-validation process. Note that the data set of RF_clim_ represents the original climate data including variables of low importance or high correlation (see section 2.3).

**Supplementary Figure 2.** Top 30 ranks of the RF_stand_ variables based on the MDG measure. The three figures show the arithmetic mean and the standard deviation of the ranking within the 10-fold cross-validation process. Note that the data set of RF_stand_ represents the original forest inventory data including variables of low importance or high correlation (see section 2.3).

**Supplementary Figure 3.** Top 30 ranks of the RF_forest_ variables based on the MDG measure. The three figures show the arithmetic mean and the standard deviation of the ranking within the 10-fold cross-validation process. Note that the data set of RF_forest_ represents the original forest inventory data including variables of low importance or high correlation (see section 2.3).

**Supplementary Figure 4.** Annual boxplot representation of the top three climate variables for *Den* as suggested by RF_VSURF_ (left panel) and RF_clim_ (right panel).

**Supplementary Figure 5.** Annual boxplot representation of the top three climate variables for *Dip* as suggested by RF_VSURF_ (left panel) and RF_clim_ (right panel).

**Supplementary Figure 6A.** Example of important forest_ variables for *Lym* represented by the density distribution of the mean Shannon evenness Black bars show the distribution for all FC in Brandenburg, red bars indicate defoliated FC.

**Supplementary Figure 6B.** Example of important forest_ variables for *Lym* represented by the mean basal area weighted stand diameter at breast height of the upper forest layers within the 1,000 ha buffer zone. Black bars show the distribution for all FC in Brandenburg, red bars indicate defoliated FC.

**Supplementary Figure 7A.** Example of important forest_ variables for *Dip* represented by the density distribution of the mean Shannon evenness. Black bars show the distribution for all FC in Brandenburg, blue bars indicate defoliated FC.

**Supplementary Figure 7B.** Example of important forest_ variables for *Dip* represented by the mean basal area weighted stand diameter at breast height of the upper forest layers within the 1,000 ha buffer zone. Black bars show the distribution for all FC in Brandenburg, blue bars indicate defoliated FC.
